# What We Stand for in Public Health

**Published:** 1923-01

**Authors:** 


### HOSPITAL and HEALTH REVIEW

### WHAT WE STAND FOR IN PUBLIC HEALTH.

"IN legislation," wrote Disraeli to Hicks Beach in December, 1874, "it is not merely reason and propriety which are to be considered, but the temper of the time." Disraeli had given the artisans the vote in 1867. What they wanted, says his biographer, and what in the General Election of 1874 they set themselves to obtain, were better, healthier, more humanising conditions in their own daily life. They demanded improvement and not revolutionary change; and, continues the biographer, to give effect to this desire, two Labour members, Alexander Macdonald and Thomas Burt, the forerunners of a mighty political force, had been returned for the first time to Parliament.

The temper of our own time has not merely returned to power a Conservative party with a comfortable majority, and with Economy and Tranquillity as watchwords, it has sent to Parliament close upon 150 members of the Labour party who may be relied upon to press the demand for social improvement, if not for revolutionary change. It would be understandable if the prolonged war-weariness, the oppressive taxation, and the exactions of foreign affairs were to lead the party returned to power to misinterpret the demand for Tranquillity as meaning a policy of Drift in regard to social legislation at home. The presence of so powerful a Labour opposition will, however, certainly provide any stimulus that may be required in a contrary direction; apart from that, a consciousness of the need for social legislation has long ceased to be the province of any particular party. In the forefront of social legislation we place health measures, and the need for "better, healthier, more humanising conditions" is just as insistent to-day as ever it was.

Let us gratefully pay tribute to the tremendous strides which have been made along the road to public health in the last fifty years—the beneficent factory legislation, the advances made in water supply, in domestic sanitation, in the control of infectious disease, and in the care of children. But let us be on our guard against marking time, and still more against set-backs, in this all-important province of health. The past ten years, owing to the war and its aftermath, cannot be reckoned amongst the years of fresh progress, even though they include the setting up of the Ministry of Health in 1919. The Ministry emerged with credit from the scrutiny of the Geddes

Committee; the late Minister of Health received almost with complacency the plaudits of the tax-payer because he had wound up the costly State housing schemes of his predecessor, without, however, putting any plan in its place. We are aware that before the war the Chancel or of the Exchequer was accused of competing with the great spending departments in the disbursement of public money; it would be, in our view, disastrous if the great department entrusted with the betterment of the public health were to become a mere annexe of the Treasury. Does anyone suppose, for instance, that the housing question can be left where it is? There is evidence, wherever we turn, that the scarcity of houses is appalling, that slum areas remain uncleared, nay, that houses considered unfit for human habitation in years before the war are no longer empty. What is being done? The local authorities may well be disappointed and display some resentment at the way in which they were first granted State housing subsidies and then given to understand that the subsidies would cease. But they cannot therefore wash their hands of the whole business. It rests with them to tackle their own housing problems, and the longer this is delayed the greater will be the bill ultimately to be met, arising from the folly of continuing breeding grounds for tuberculosis and other diseases. Housing is undoubtedly the most urgent of the problems in the field of public health, calling for the unremitting attention of the local authorities. This is not to say that they ought to be left to deal with this costly and vital problem single-handed. Neither the State nor the municipalities, nor the two combined, can hope to make good the arrears of housing and to maintain the steady flow of new homes annually demanded by the natural growth of the population. We must once more look to private enterprise, which before the war provided all but a fraction of the large output of new dwellings, to perform its proper economic function, which it will not do if it is legislatively interfered with. But the plan, the organisation, the driving force, must come from the local authorities, with the stimulus of the Ministry of Health behind them.

Although housing is the most important, most urgent, and most far-reaching problem connected with the public health, there is a wide field for constant vigilance, for experiment, for research, for education and propaganda. There is need for a constant supply of information of what other authorities are doing, and for a healthy spirit of competition between localities. There is need for a continual quickening of the public conscience. There is need also for economy. But it is not the primary function of the member of a Public Health Committee to approach a demand for public health expenditure with the question: Will this involve a penny or twopence on the rates? There will be plenty of voices to raise this question; it is for the member of the Public Health Committee to look into the need with the help of the Council's medical adviser, and on being satisfied that it is a real and urgent necessity firmly press it forward.

In that great classic, "English Sanitary Institutions," the late Sir John Simon says, "Sanitary laws and sanitary administration mean to me laws and administration for the saving and strengthening of life." They are, he says, of the province where Medicine joins hands with Common Sense, and "to procure for the life and happiness of the nation the utmost possible freedom from interruptions by disease is a task well worthy to engage the best energies of many best minds." Elsewhere, in his appeal to educated local patriotism, he refers to service on local councils as that which should commend itself more and more as an object of legitimate and generous ambition to "persons of good business ability and sufficient leisure who would have in view no other purpose than that of being actively useful in public duty."

We make particular appeal in these columns to members of the Public Health Committees who are in that position because they are genuinely zealous for the health of the community in which they dwell. The first step in the direction of any reform is full and accurate information, and we hope that members of Public Health Committees and their medical officers will co-operate with us in moulding and enlightening public opinion by furnishing us with accounts of special features in their health administration, and of special steps in any direction which they may make experimentally or in advance of other authorities, and thus to put individual experience into the common pool. The primary object of this journal—apart from its long-standing interest in the hospitals—is the betterment of the public health and the creation of a sound public opinion upon the many subjects included in that comprehensive phrase. This object can only be attained by the co-operation to which we have referred. Our aim is that The Hospital and Health Review shall be at once a forum and a clearing-house—a forum for the free and frank discussion of every subject bearing, directly or indirectly, upon the health of the nation, and a clearing-house for ideas and suggestions emanating from a great variety of sources at home and abroad. The betterment of the public health is not sacred to medical or other experts, though we must ever look to them for scientific guidance. It is the concern of us all, and especially of the great body of laymen who accept responsibility for the public health of their county or town. It is for them to see that their existing powers are used to the uttermost, and that where those powers are inadequate for the proper discharge of their functions as custodians of the public health, their collective views and experience should be pressed on the Government, and the additional powers obtained. We have referred at the beginning of this article to Disraeli. It was he also in 1874 who spoke of sanitary reform as embracing most of the civilising influences of humanity, and who passed, in the following year, that Public Health Act which formed the foundation of our present sanitary code. He expressed in a brilliant phrase the fact of the public mind being exercised in regard to the public health; we commend it to the Government as a New Year's motto—*Sanitas sanitatum, omnia sanitas.* No Government, when it comes to render its accounts, could desire a nobler epitaph.

